# Automatic volumetric diagnosis of hepatocellular carcinoma based on four-phase CT scans with minimum extra information

**DOI:** 10.3389/fonc.2022.960178

**Published:** 2022-10-13

**Authors:** Yating Ling, Shihong Ying, Lei Xu, Zhiyi Peng, Xiongwei Mao, Zhang Chen, Jing Ni, Qian Liu, Shaolin Gong, Dexing Kong

**Affiliations:** ^1^ School of Mathematical Sciences, Zhejiang University, Hangzhou, China; ^2^ Department of Radiology, The First Affiliated Hospital, College of Medicine, Zhejiang University, Hangzhou, China; ^3^ Computational Imaging and Digital Medicine, Zhejiang Qiushi Institute of Mathematical Medicine, Hangzhou, China; ^4^ Department of Radiology, The Hospital of Zhejiang University, Hangzhou, China; ^5^ Department of Radiology, Jiangcun Community Health Service Center, Hangzhou, China

**Keywords:** computed tomography, diagnosis, hepatocellular carcinoma, deep learning, arificial intelligence

## Abstract

**Summary:**

We built a deep-learning based model for diagnosis of HCC with typical images from four-phase CT and MEI, demonstrating high performance and excellent efficiency.

**Objectives:**

The aim of this study was to develop a deep-learning-based model for the diagnosis of hepatocellular carcinoma.

**Materials and methods:**

This clinical retrospective study uses CT scans of liver tumors over four phases (non-enhanced phase, arterial phase, portal venous phase, and delayed phase). Tumors were diagnosed as hepatocellular carcinoma (HCC) and non-hepatocellular carcinoma (non-HCC) including cyst, hemangioma (HA), and intrahepatic cholangiocarcinoma (ICC). A total of 601 liver lesions from 479 patients (56 years ± 11 [standard deviation]; 350 men) are evaluated between 2014 and 2017 for a total of 315 HCCs and 286 non-HCCs including 64 cysts, 178 HAs, and 44 ICCs. A total of 481 liver lesions were randomly assigned to the training set, and the remaining 120 liver lesions constituted the validation set. A deep learning model using 3D convolutional neural network (CNN) and multilayer perceptron is trained based on CT scans and minimum extra information (MEI) including text input of patient age and gender as well as automatically extracted lesion location and size from image data. Fivefold cross-validations were performed using randomly split datasets. Diagnosis accuracy and efficiency of the trained model were compared with that of the radiologists using a validation set on which the model showed matched performance to the fivefold average. Student’s *t*-test (T-test) of accuracy between the model and the two radiologists was performed.

**Results:**

The accuracy for diagnosing HCCs of the proposed model was 94.17% (113 of 120), significantly higher than those of the radiologists, being 90.83% (109 of 120, *p*-value = 0.018) and 83.33% (100 of 120, *p*-value = 0.002). The average time analyzing each lesion by our proposed model on one Graphics Processing Unit was 0.13 s, which was about 250 times faster than that of the two radiologists who needed, on average, 30 s and 37.5 s instead.

**Conclusion:**

The proposed model trained on a few hundred samples with MEI demonstrates a diagnostic accuracy significantly higher than the two radiologists with a classification runtime about 250 times faster than that of the two radiologists and therefore could be easily incorporated into the clinical workflow to dramatically reduce the workload of radiologists.

## Highlights

1. The accuracy for diagnosing hepatocellular carcinomas of the proposed model and two radiologists was 94.17% (113 of 120), 90.83% (109 of 120, *p* = 0.018), and 83.33% (100 of 120, *p* = 0.002), showing significant differences.
2. The average time analyzing each lesion by our proposed model was 0.13 s, which was hundred times faster than the two radiologists.
3. The proposed model can serve as a quick and reliable “second opinion” for radiologists.

## Introduction

Hepatocellular carcinoma (HCC) is the third most common malignancy worldwide, with incidence rates continuing to rise (1). CT slices often serve as an important assistive diagnostic tool for HCCs (2). According to the American Association for the Study of Liver Disease (AASLD) and the Liver Imaging Reporting and Data System (LI-RADS) reported by the American College of Radiology, the hallmark diagnostic characteristics of HCC on multi-phasic CT slices are arterial phase hyper-enhancement followed by washout appearance in the portal-venous and/or delayed phases (3, 4). Four-phase CT slices that contain non-enhanced, arterial, portal-venous, and delayed phases are recommended as the clinical standard. However, ensuring the diagnosis performance of a computer-aided system equivalent to that of radiologists with minimum extra information (MEI) about the patients for instance including only basic data about age and gender on a relatively small dataset based on four-phase CT images is still challenging in order to relieve the radiologists’ workload as well as to improve the diagnosis throughout (5).

Machine learning algorithms have been widely applied in the radiological classification of various diseases and may potentially address this challenge (6–8). Recently, among different machine learnings, deep learning with convolutional neural network (CNN) have achieved state-of-the-art performances with respect to pattern recognition of images for various organs and tissues (9–15). It has been verified that CNN-based methods show high diagnostic performance in differentiation of tumors (16–20), but with most of them being limited to 2D slices, which needs manual selection. Meanwhile, it does not take advantage of 3D information that can potentially improve the diagnostic performances (21–25). Moreover, previous works (16, 17, 19, 25) for liver tumor diagnosis use three-phase CT slices, namely, non-enhanced phase, arterial phase, and transitional phase, which is between the portal-venous phase and the delay phase. However, hypointensity in the transitional phase does not qualify as “washout”, which is considered a strong predictor and major criterion of HCC (3, 4). Therefore, in this study, we propose a 3D residual network (ResNet) as our basis network to explore the 3D structural information with four-phase CT images for tumor diagnosis (26).

Typically, high-performing CNN requires training on large datasets, which unfortunately are difficult to obtain especially in the medical field. As an alternative to large datasets, highly complicated clinical data collected from multi-modalities are incorporated to the CNN models (27, 28). Numerous works have discussed the auxiliary role of clinical data for HCC diagnosis, including, for example, alpha fetoprotein as a serological marker for HCCs since the 1960s (29), hepatitis B virus infection (30), and medical record of having non-alcoholic fatty liver diseases (31). However, those clinical data often require additional examinations. Therefore, it would be better if one only needs the patient’s basic information, such as age and gender, which is crucial for liver tumor diagnosis (32–34) and makes full use of the spatial morphological information of local lesions that may be lost or downplayed in image processing.

In summary, our study aims to develop a fast-processing deep learning algorithm that exploits 3D structural with dynamic contrast information from four-phase CT scans and requires minimum patient information, i.e., age and gender, as well as automatically extracted lesion location and size from image data based on a relatively small dataset. We name the algorithm as the MExPaLe model (Model Fused with Minimum Extra Information about Patient and Lesion). The main contributions of this work are as follows:

- We propose a 3D model that feeds volumetric data as input instead of 2D CT slices to improve the diagnosis performance.
- We evaluate the diagnosis results of the basic model, which only uses non-enhanced phase CT images as input and enhanced model, which adds contrast-enhanced phase images as additional inputs. We experimentally confirm the necessity of using enhanced contrast agents in clinical workflow.
- The MExPaLe model fuses CNN and multilayer perceptron to incorporate two different modalities: image data and text data. The text data contain only information of patient gender and age, appended with the spatial morphological information of local lesions.
- The MExPaLe model demonstrates high performance and excellent efficiency. The accuracy and time efficiency for liver diagnosis of the proposed model are significantly higher than the two radiologists.

This paper is organized into four sections. In *Section 2*, we first describe the data collected in our paper, then introduce three models in this study, and finally the evaluation metrics have been presented. *Section 3* presents the results of our models and the comparison with other models and two radiologists. The discussion is provided in *Section 4*.

## Materials and methods

**Figure 1:**
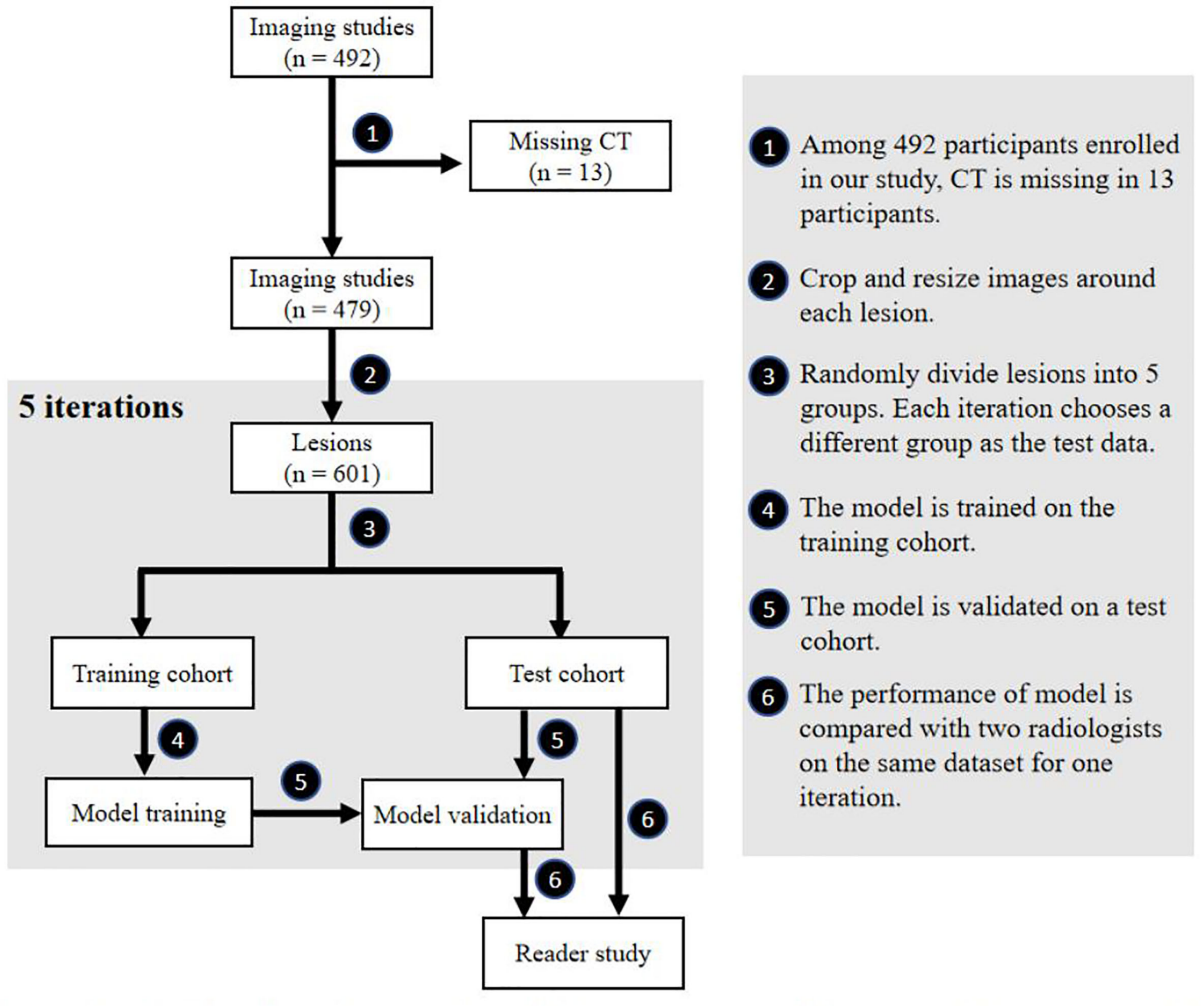
Flowchart of our study. Participant selection, model training, model testing and reader study are included in our study.

This retrospective clinical study was approved by the review board, and the requirement for written informed consent was waived. Patients diagnosed as benign and HA through 1-year follow-up in 2018 while diagnosed as HCC and ICC after surgery or biopsy were enrolled between 2014 and 2017. Individuals without four-phase CT images were excluded, shown in **Figure 1**. Ultimately, a total of 601 lesions (315 HCCs) from 479 patients were selected. The details are presented in **Table 1**.

**Table 1:** Patient characteristics and demographics.

Patient characteristics	HCC	Cyst	HA	ICC	Total
Number of patients	312	37	107	41	479
Number of lesions	315	64	178	44	601
Age at imaging (mean ± std)	58 ± 11	58 ± 7	50 ± 10	59 ± 10	56 ± 11
Gender					
Male	268	28	41	29	350
Female	44	9	66	12	129

HCC, hepatocellular carcinoma; HA, hemangioma; ICC, intrahepatic cholangiocarcinoma; std, standard deviation.

### Data preprocessing

All CT slices were obtained with PHILIPS Brilliance iCT 256 scanner (Philips Healthcare, Netherlands). Contrast enhancement materials (Ultravist 300-3440, Bayer Schering Pharma AG, Germany) were injected. These four-phase CT images, stored as DICOM files, have a size of 512×512, and the thickness of each slice is 3 or 5 mm. The target lesions were manually labeled with 3D bounding boxes by a radiologist with 10 years of experience (XM) using software designed by Peng et al. (35) and revised if needed by a radiologist with 38 years of experience (ZY). The images were further processed by code written in the programming language Python 3.6 (https://www.python.org). We first reshaped the four-phase images to 1×1×1 mm using the cubic spline interpolation method and extracted the lesions and the surrounding 5-mm pixels by the bounding boxes. Then, the cropped 3D images were resized to a resolution of 64×64×64 voxels. The images were finally randomly selected to comprise the test data using fivefold cross-validation with the remaining images being the training data. **Table 2** summarizes the distribution of each experiment.

**Table 2:** Distribution of the fivefold cross-validation dataset.

Experiment	E1	E2	E3	E4	E5
Training data	480	481	481	481	481
HCC	252	252	252	252	252
Non-HCC	228	229	229	229	229
Test data	121	120	120	120	120
HCC	63	63	63	63	63
Non-HCC	58	57	57	57	57
Total	601	601	601	601	601

HCC, hepatocellular carcinoma; E1–E5 denote five sets of experiments.

The gender and age of the patients are the basic information recorded in the clinical system. Their contributions to HCC and non-HCC including benign, HA, and ICC diagnosis were evaluated in this study. In addition, the location and size of the lesions are inevitably lost during the common data preprocessing procedure. Therefore, we recorded the maximum normalized size and the relative location of the bounding box as our spatial morphological information during the data preprocessing. We also evaluated the contribution of spatial morphological information for HCC diagnosis.

### Models

**Figure 2:**
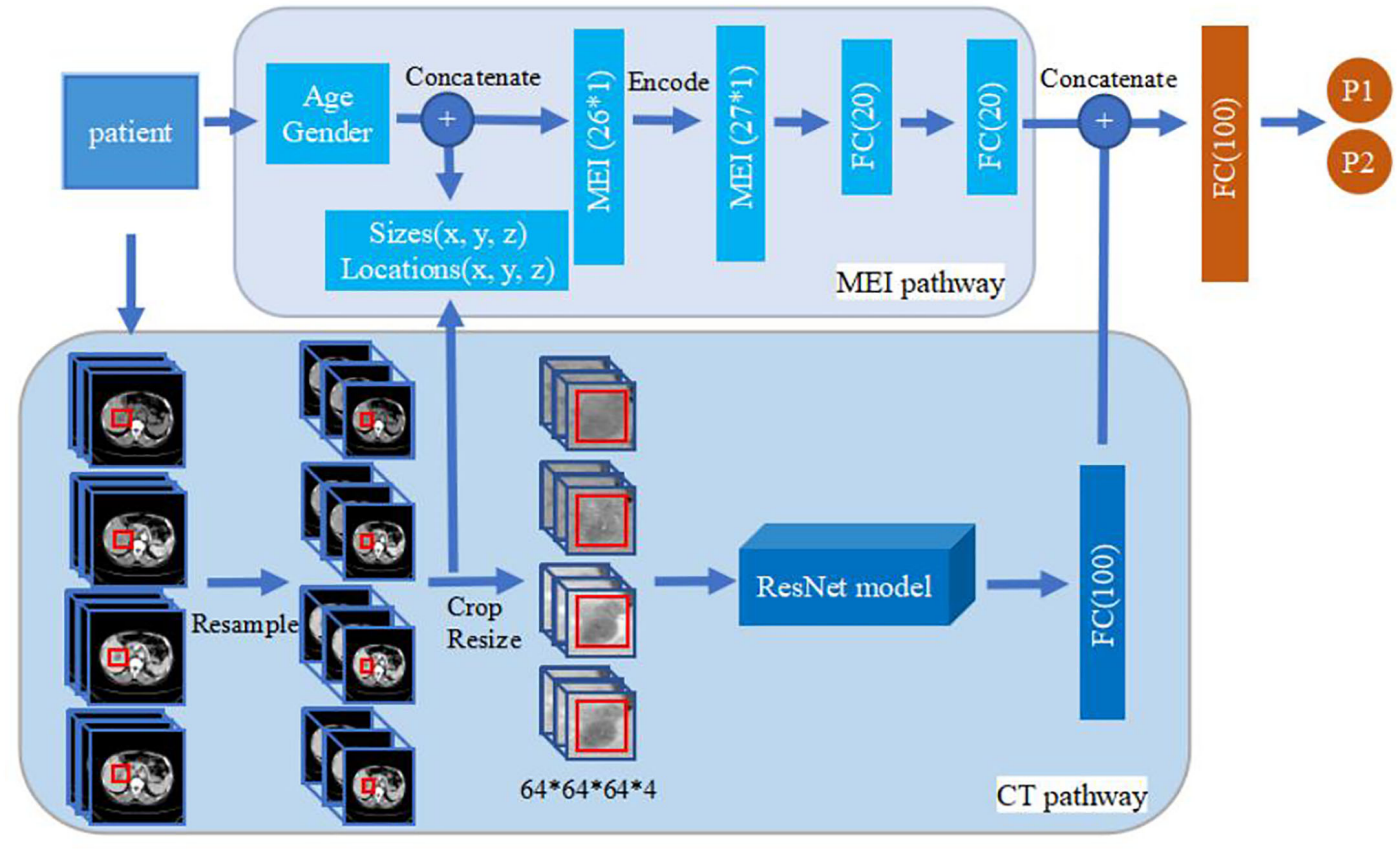
Overview of the proposed method. The upper part is MEI pathway and the lower part is the CT pathway. The 3D ResNet in CT pathway contains 14 layers (13 convolution layers, and 1 global average pooling layer). Filter size of the first convolution layer is 5×5×5, and the following filter sizes are 3×3×3. Filter size of the global average pooling layer is 2×2×2. The basic model and enhanced model only have the CT pathway. The size of image input in basic model is 64×64×64×1 while the others are 64×64×64×4. MEI, Minimum Extra Information.

The model was built using Keras 2.2.4 (https://keras.io/) with a Tensorflow backend 1.5.0 (https://www.tensorflow.org/). For a baseline, we built a deep learning model based on the structure of 3D ResNet with 14 layers (13 convolutional layers and 1 global average pooling layer). Filter size of the first convolution layer is 5×5×5, and the following filter sizes are 3×3×3. The filter size of the global average pooling layer is 2×2×2. The basic model only uses non-enhanced phase CT images as input while the enhanced model adds contrast-enhanced phase images as additional input. For the basic and enhanced model, a fully connected layer is added following the 3D ResNet structure, whose output value represents the probability belonging to the corresponding class. The MExPaLe model contains two pathways: the CT pathway and the MEI pathway. The CT pathway has the same design as the aforementioned 3D ResNet structure but with the final classification layer removed. The MEI contains the patient age and gender exacted from the DICOM files and the relative size and location of lesions exacted from the CT pathway. MEI is text information; thus, we used a multilayer perceptron model containing two fully connected layers for this pathway. In our model, after the high-level features are flattened, image features and the text features are concatenated together. Finally, the concatenated feature vector is connected to a fully connected layer for final classification. The overview of the proposed method is shown in **Figure 2**.

All models use rectified linear units to help models learn non-linear features. These are used in conjunction with batch normalization and dropout to reduce overfitting. Each model was trained with a stochastic gradient descent optimizer using minibatches of eight samples. Each model was trained for 80 epochs. The training rate was initially set to 0.01, and it was reduced by half every 10 epochs.

The performance of the MExPaLe model was compared with two certified radiologists. The two radiologists (HY, with 21 years of imaging experience, and HC, with 16 years of imaging experience) did not take part in the data annotation process and were blinded to the lesion selection. For fair comparison and to simultaneously mimic the real working scenario as closely as possible, we provided four-phase CT DICOM data and the corresponding lesion 3D bounding boxes to both the MExPaLe model and radiologists. The test set for the reader study consisted of 120 randomly selected lesions in total (63 HCCs), while the remaining lesions were assigned to the training set. The time for the model from reading CT phases until classification of the lesion was recorded.

### Statistics

Receiver operating characteristic (ROC) analyses were performed to calculate the area under curve (AUC) for evaluating model performance. The average accuracy, sensitivity, specificity, positive predictive value (PPV), and negative predictive value (NPV) for diagnosing each category were calculated. Student’s *t*-test (T-test) using IBM SPSS Statistics 26.0 was also performed to evaluate the statistical significance of differences in comparative studies.

## Results

**Figure 1** shows the flowchart of our study, including participant selection, model training, model testing, and reader study. A total of 479 participants (350 men and 129 women) were enrolled in our study. The mean age ± standard deviation at enrollment was 56 years ± 11. Summaries of included participants are described in **Table 1**.

### Basic model and enhanced model

The diagnosis performances of the basic model and the enhanced model are shown in **Table 3**. Compared with the basic model, the enhanced model shows higher accuracy (17.30% higher in average, 91.68% *vs*. 74.38%, *p* < 0.001), AUC (18.47% higher in average, 95.79% *vs*. 77.32%, *p* < 0.001), sensitivity (12.06% higher in average, 94.60% *vs*. 82.54%, *p* = 0.029), specificity (23.03% higher in average, 88.45% *vs*. 65.42%, *p* = 0.001), PPV (17.34% higher in average, 90.03% *vs*. 72.69%, *p* < 0.001), and NPV (15.45% higher in average, 93.77% *vs*. 78.32%, *p* = 0.008).

**Table 3:** Performance of basic model and enhanced model.

Parameter (%)	Basic model	Enhanced model	*p*-value^*^
Accuracy	74.38 (70.25–80.00)	91.68 (86.67–95.87)	**< 0.001**
AUC	77.32 (69.08–83.65)	95.79 (92.93–98.09)	**< 0.001**
Sensitivity	82.54 (69.84–95.24)	94.60 (90.47–100.00)	**0.029**
Specificity	65.42 (53.45–95.24)	88.45 (82.46–91.38)	**0.001**
PPV	72.69 (66.67–76.92)	90.03 (85.07–92.64)	**< 0.001**
NPV	78.32 (68.85–92.31)	93.77 (88.67–100.00)	**0.008**

AUC, area under curve; PPV, positive predictive value; NPV, negative predictive value. Data are median values in brackets and range in parentheses.

*p-value for differences between basic model and enhanced model, calculated with Student’s t-test.

The bold values show the significant differences between basic model and enhanced model.

**Figure 3:**
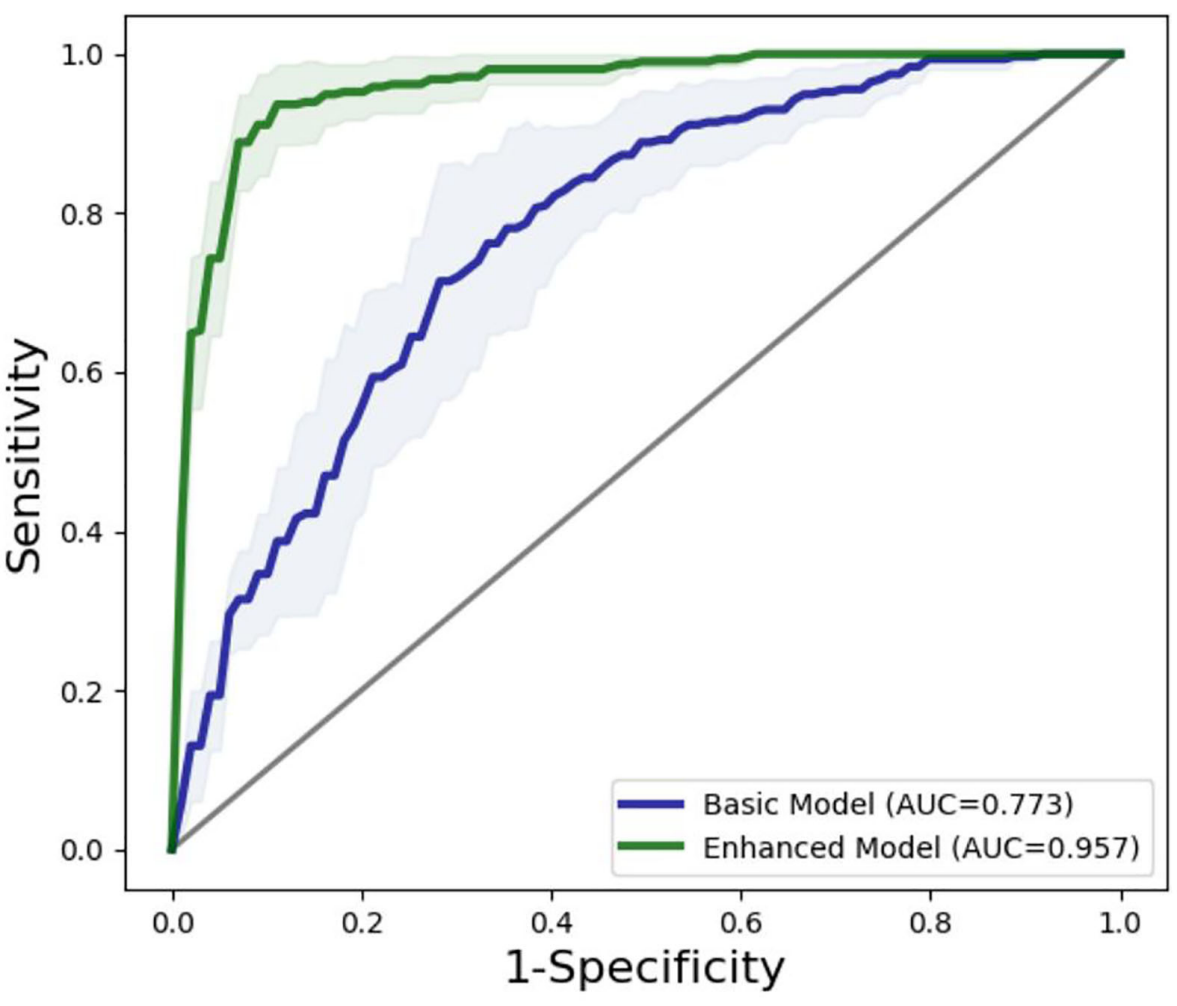
ROC curves of basic model and enhanced model. The lines reflect the average performances of the models, and the light-colored area reflects the fluctuation of the models represented by the corresponding standard deviations.

**Figure 4:**
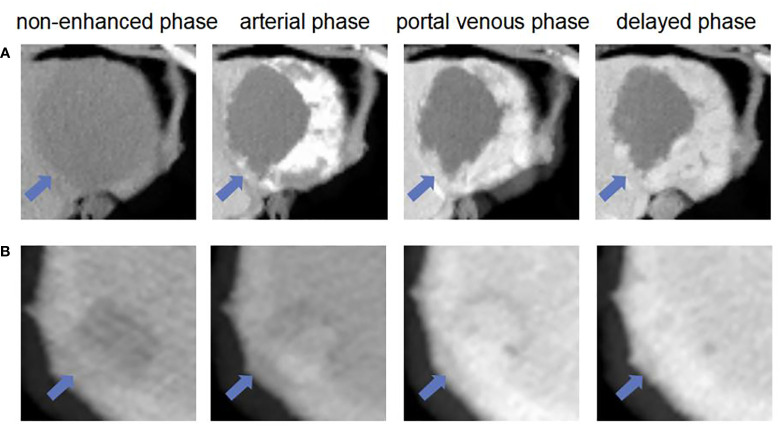
The liver masses misdiagnosed by models. **(A)** shows four phase images of a 62-year-old man with a hemangioma (arrow) that was diagnosed through one-year follow-up in 2018. The mass was correctly diagnosed as non-HCC by using enhanced model and our MExPale model. It was misdiagnosed as HCC by using basic model. **(B)** shows four phase images of 54-year-old man with a HCC (arrow) that was diagnosed after surgery. The mass was correctly diagnosed as HCC by using our MExPale model. It was misdiagnosed as HCC by using basic model and enhanced model.

The ROC curves of the basic and enhanced models with the corresponding AUC values are shown in **Figure 3**. The liver masses misdiagnosed by the basic model or enhanced model are shown in **Figure 4**. We present four-phase images of a 62-year-old man with a hemangioma and a 54-year-old man with an HCC. The major criterion of HCC such as “wash out” cannot be extracted by the model without the contrast-enhanced CT slices, which leads to the poor performance of the basic model.

### MExPaLe model

**Figure 5:**
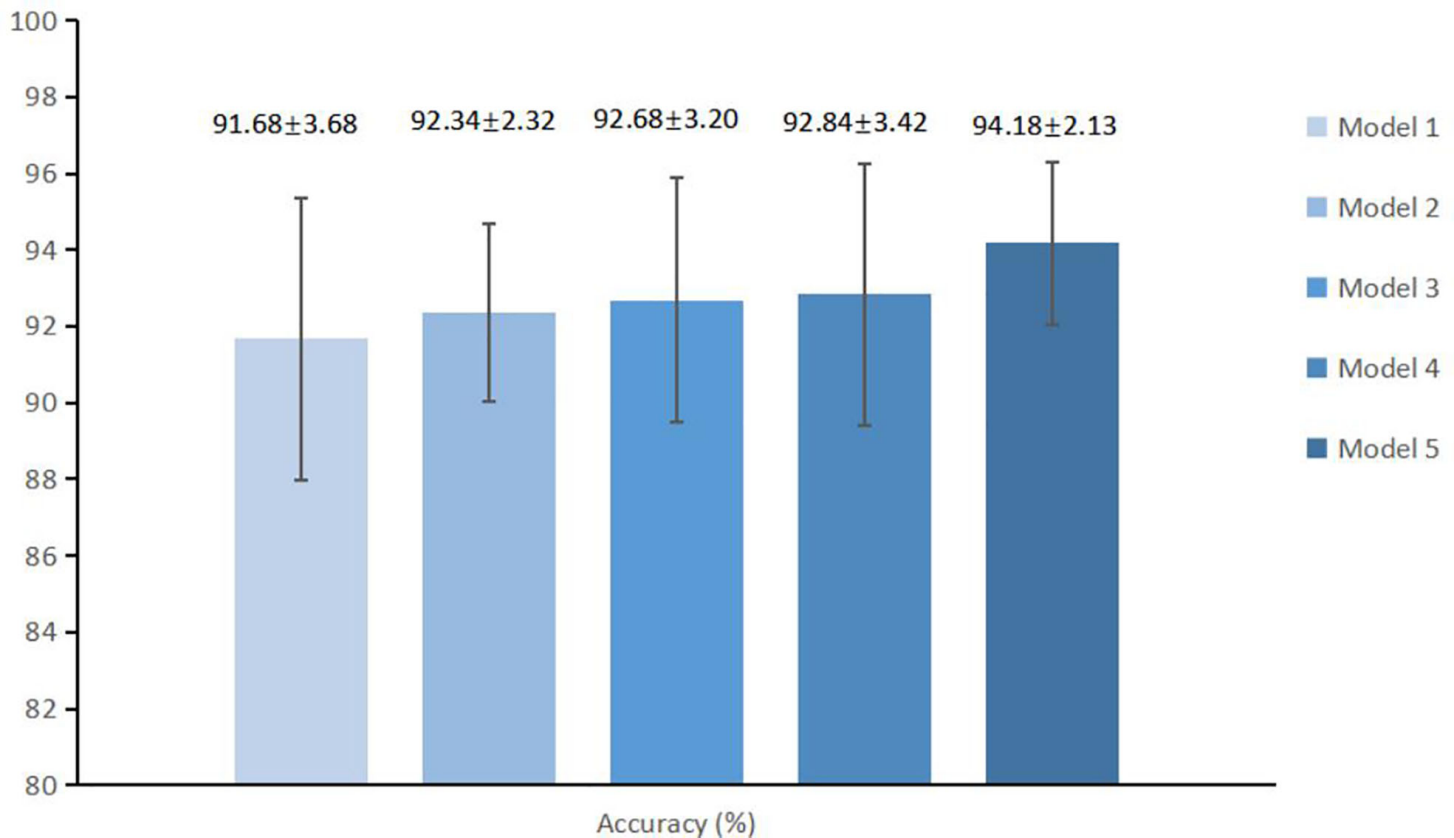
The average accuracy and standard deviations of different models. Model 1, Enhanced model; Model 2, Enhanced model with spatial morphological information; Model 3, Enhanced model with spatial morphological information and age; Model 4, Enhanced model with morphological information and gender; Model 5, MExPale model.

In order to further improve the diagnosis, we first extracted the spatial morphological information of the local tumor during the data preprocessing process. Then, we added the patient’s age and gender information, which were automatically recorded in the medical system. We finally compared the average diagnosis accuracy of models with different extra information, as shown in **Figure 5**. The average accuracy of the MExPaLe model was 94.18%, which was higher than that of the enhanced model (91.68%), the enhanced model with spatial morphological information (92.34%), the enhanced model with spatial morphological information and age (92.68%), and the enhanced model with spatial morphological information and sex (92.84%).

**Figure 6:**
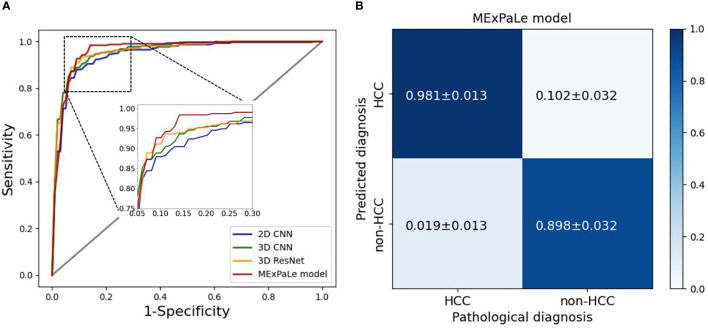
Performance of models. **(A)** ROC curves of models, **(B)** The confusion matrix of our MExPale model. HCC, hepatocellular carcinoma.

The diagnostic performance of the MExPaLe model compared with other authors is shown in **Table 4**. The MExPaLe model achieved an average accuracy of 94.18%, which was 4.99% higher than 2D CNN, 3.34% higher than 3D CNN, and 2.50% higher than 3D ResNet. Particularly, the MExPaLe model showed good performance in terms of specificity and NPV. The ROC curves of models are described in **Figure 6A**, and the confusion matrix of the MExPaLe model is described in **Figure 6B**. The MExPaLe model achieved an average AUC of 96.31%, which was 1.53% higher than 2D CNN, 0.31% higher than 3D CNN, and 0.52% higher than 3D ResNet. The average ratio of true positive was 98.10%, and the average ratio of true negative was 89.85%.

**Table 4:** Performance of models.

Parameter (%)	Accuracy	AUC	Sensitivity	Specificity	PPV	NPV
2D CNN (16)	89.19	94.78	89.21	89.17	90.11	88.30
3D CNN (16)	90.84	96.00	91.75	89.84	90.90	91.06
3D ResNet (25)	91.68	95.79	94.60	88.45	90.03	93.77
**MExPaLe model**	**94.18**	**96.31**	**98.10**	**89.85**	**91.45**	**97.70**

AUC, area under curve; PPV, positive predictive value; NPV, negative predictive value. The 3D CNN is generated from 2D CNN in (16).

The bold values show the best performance of models.

### Reader study

**Figure 7:**
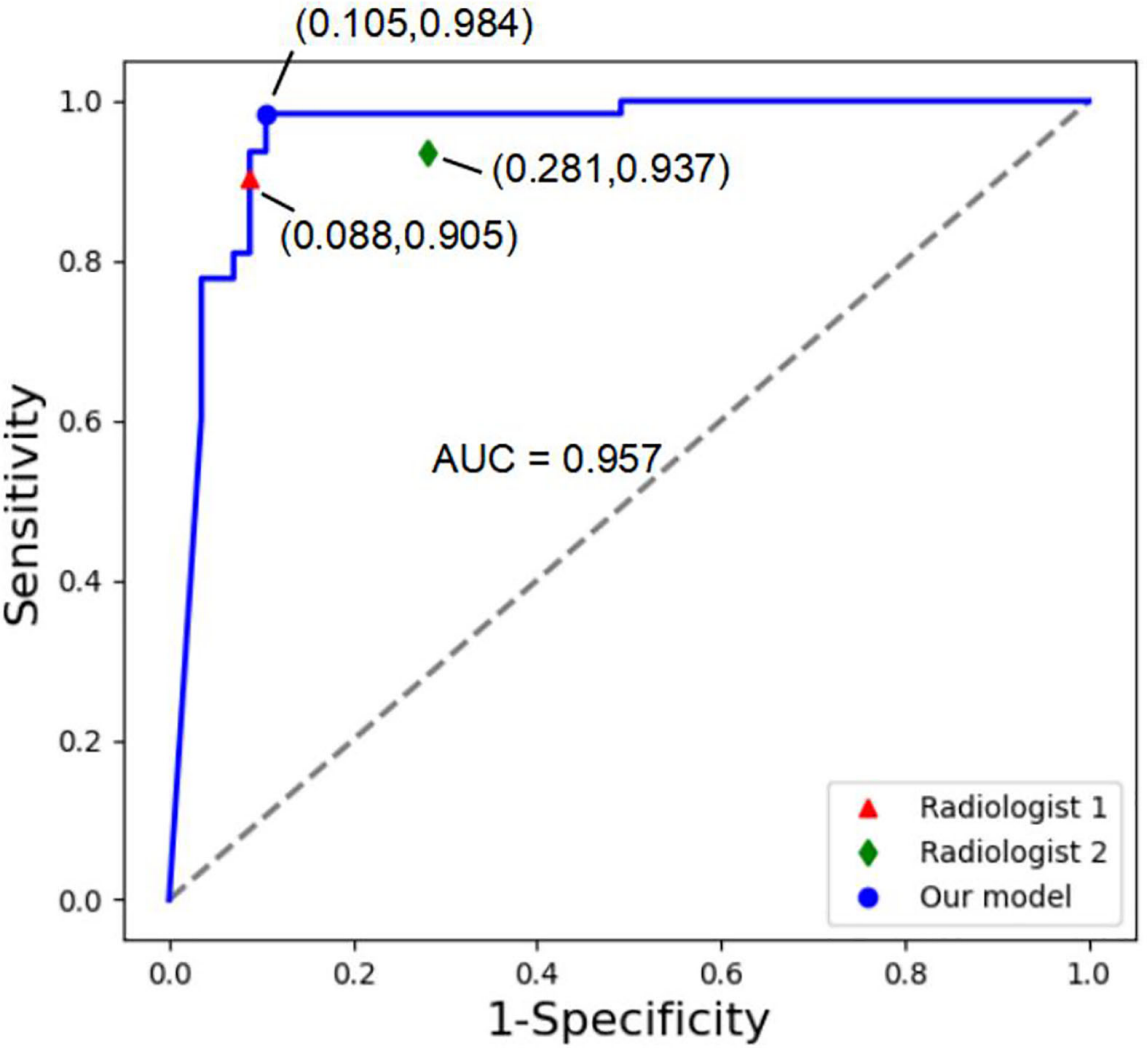
ROC curves of our MExPale model and two radiologists. Radiologist 1 comes from the First Affiliated Hospital of Zhejiang University, and Radiologist 2 comes from a community primary hospital.

**Figure 8:**
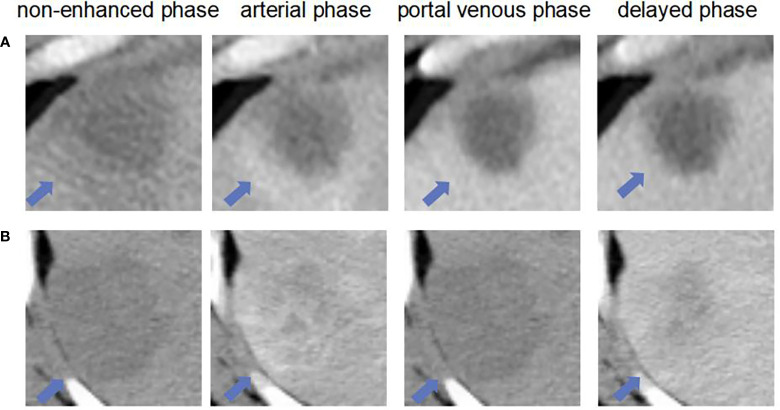
The liver masses misdiagnosed by model and two radiologists. **(A)** shows four phase images of a 59-year-old man with a HCC (arrow) that was diagnosed after surgery. The mass was misdiagnosed diagnosed as non-HCC by and our MExPale model and both two radiologists. **(B)** shows four images of a 64-year-old man with a ICC (arrow) that was diagnosed after surgery. The mass was misdiagnosed diagnosed as HCC by our MExPale model and both two radiologists.

In the reader study, classification of 120 randomly selected lesions by the MExPaLe model achieved an accuracy of 94.17% (113/120). Diagnosis accuracies by radiologists from the First Affiliated Hospital of Zhejiang University (radiologist 1) and from the community primary hospital (radiologist 2) on the same lesions were 90.83% (109/120) and 83.33% (100/120), respectively (**Table 5**). We then randomly divided the lesions into five equal parts using T-test for statistical comparisons between the radiologists and our proposed MExPaLe model. The *p*-values comparing the MExPaLe model and radiologists 1 and 2 were 0.018 and 0.002, respectively, suggesting significant differences. The average runtime analyzing each lesion was 0.13 s for the MExPaLe model on one Graphics Processing Unit, while for the radiologists, on average 30 s and 37.5 s were needed. ROC curves of our MExPaLe model and two radiologists are shown in **Figure 7**. The misdiagnosed cases of the model and radiologists are described in **Table 6**. The coincidence degree between the MExPaLe model and radiologist 1 was 16.67% for HCC masses and 10.00% for non-HCC masses, while with radiologist 2, the coincidence degree was 25.00% for HCC masses and 22.22% for non-HCC masses. Our model showed a lower misdiagnosis rate for HCC masses compared with the two radiologists. Moreover, the performance of our model was more stable than those of the radiologists, with radiologist 1 showing high misdiagnosis for HCC masses and radiologist 2 showing high misdiagnosis for non-HCC masses. Some representative masses with varying diagnostic results from the MExPaLe model and the two radiologists are shown in **Figure 8**. As shown in **Figure 8B**, 71.43% (5/7) of the misdiagnosed cases by the model were ICC masses being misdiagnosed as HCC masses. This also constitutes the majority of misdiagnoses by the radiologists since it is hard to differentiate HCC from ICC especially owing to the low incidence rate of ICC. Therefore, by increasing the cases of ICC to balance the dataset, the model performance can be improved in the future.

**Table 5:** Overall accuracy and times for model and radiologists’ classification.

Parameter	MExPaLe model	Radiologist 1	*p*-value^*^	Radiologist 2	*p*-value^*^
Accuracy (%)	**94.17 (113/120)**	90.83 (109/120)	0.018	83.33 (100/120)	0.002
Time	**0.13 s**	30 s	–	37.5 s	–

Radiologist 1 comes from the First Affiliated Hospital of Zhejiang University, and Radiologist 2 comes from a community primary hospital.

*p-value for differences between the MExPaLe model and radiologists, calculated with Student’s t-test. The bold values show the best performance in terms of accuracy and time.

**Table 6:** Misdiagnosed images for model and radiologists’ classification.

Parameter	MExPaLe model	Radiologist 1	Radiologist 2
Misdiagnoses			
HCC	**1**	6	4
Non-HCC	**6**	5	16
Coincidence degree			
HCC	-	16.67% (1/**6**)	25.00% (1/**4**)
Non-HCC	–	10.00% (1/**10**)	22.22% (4/**6**)

Radiologist 1 comes from the First Affiliated Hospital of Zhejiang University, and Radiologist 2 comes from a community primary hospital. HCC, hepatocellular carcinoma.

The bold values mean the number of misdiagnosed masses for our model classification.

## Discussion

In this work, we built a deep learning-based model, MExPaLe, for the diagnosis of liver tumor with typical images from four-phase CT and MEI, demonstrating high performance and excellent efficiency. The accuracy for diagnosing liver tumors of the proposed model and the two radiologists were 94.17% (113 of 120), 90.83% (109 of 120, *p* = 0.018), and 83.33% (100 of 120, *p* = 0.002), showing significant differences. The average time analyzing each lesion by our proposed MExPaLe model was 0.13 s, which was close to 250 times faster than that of both radiologists.

We used volumetric 3D CT patches as inputs. The 3D model can provide more relevant information to lesion classification, minimizing model variability, and it was not dependent on manual slice selection. Concerns for using the 3D model may involve possible expensive computational cost and time consumption. However, by focusing on local liver lesions and a relatively shallow model structure, we achieved sub-second runtime per case, taking four-phase CT volumetric scans as input, and therefore, it no longer becomes a practical obstacle.

In real clinical conditions, critical diagnostic features, such as hyper-enhancement and washout, are the main features used by radiologists. These features are obtained through the comparison of multi-phase CT images, necessitating the use of enhanced contrast agents to improve the diagnosis accuracy. This is also verified by our results obtained from the basic model and enhanced model, which had a median accuracy of 74.38% (range, 70.25%–80.00%) and 91.69% (range, 86.67%–95.87%), respectively, and by the statistical test.

Many works have confirmed that clinical data about the patients can improve the performance of diagnosis. However, the clinical data used in those works are often too complicated to obtain, and their processing requires additional manpower and material resources. More importantly, some clinical data can be inaccurate at the time of collection, such as family genetic history. Instead, our experiment requires only the basic information of the patient, i.e., age and gender, and minimal spatial morphological information lost during image preprocessing, which does not increase the clinical workload; therefore, it is of high practical value to be used in the clinics. The proposed MExPaLe model showed a median accuracy of 94.18% (range, 91.67%–96.67%) and a median AUC of 96.31% (range, 93.34%–98.22%). The MExPaLe model showed high specificity and NPV, attributed to the usefulness of the MEI in predicting liver tumor, which made the MExPaLe model more effective than others.

Furthermore, the MExPaLe model differs from previous works in that it does not require complex-shaped ROI tracing boundaries of tumors. The location and size of a 3D bounding box around the target lesion are enough in our work. We included 5-mm extra pixels surrounding the lesions to learn more peri-tumoral information, which is necessary for enhancing tumor differentiation. Additionally, it can reduce the possible subjective bias in the image capture process and maintain tumor size information to a certain extent.

The direct comparison between the MExPaLe model and the two radiologists suggests that the MExPaLe model can serve as a reliable and quick “second opinion” for radiologists. In the diagnosis of HCCs, the accuracy of the MExPaLe model was higher than that of the chief radiologist at a first-tier research hospital and the radiologist from a community primary hospital, both with statistical significances. Furthermore, the runtime of the MExPaLe model per case for liver tumor diagnosis was close to 250 times faster compared with the radiologists, suggesting that the use of the MExPaLe model can greatly improve the diagnosis throughput in the clinics.

While these results are promising, several limitations should be acknowledged regarding this study. Because of the limited number of imaging studies, we were restricted to a cross-validation experimental design. It would be better if we can incorporate an additional test dataset, and ideally an external dataset to consolidate the usefulness of our model in the clinical diagnosis of HCCs. Another limitation is that only four typical primary liver cancer types were available with the exclusion of other relevant cancers types including metastatic liver cancers.

In conclusion, we proposed a model for the diagnosis of liver tumor. The MExPaLe model, which has incorporated four-phase CT volumes and the MEI, achieves the highest prediction accuracy of 94.18% (range, 91.67%–96.67%) and an AUC of 96.31% (range, 93.34%–98.22%). It is superior to both the basic model and the enhanced model. It is about 250 times more time-efficient compared with the radiologists for liver tumor diagnosis, taking only 0.13 s. The architectural design of the MExPaLe model may be applicable to more multi-phase CT-based diagnosis projects to provide high-quality patient care in a time-efficient manner.

## Data availability statement

The raw data supporting the conclusions of this article will be made available by the authors, without undue reservation.

## Ethics statement

This study was reviewed and approved by Clinical Research Ethics Committee of the First Affiliated Hospital, Zhejiang University School of Medicine. Written informed consent to participate in this study was provided by the participants’ legal guardian/next of kin.

## Author contributions

Literature research, YL. Project supervision, DK. Data annotation, ZP and XM. Experiment, YL. Clinical studies, YL, LX, ZP, XM, SG, ZC, JN, QL and SY. Data analysis, YL, LX, ZC, JN, QL and SY. Statistical analysis, YL. Manuscript writing, YL. Manuscript revision, LX, YL contributed equally to this work with SY. All authors contributed to the article and approved the submitted version.

## Funding

This work was supported by the National Natural Science Foundation of China, Grant Nos. 12090020 and 12090025.

## Conflict of interest

The authors declare that the research was conducted in the absence of any commercial or financial relationships that could be construed as a potential conflict of interest.

## Publisher’s note

All claims expressed in this article are solely those of the authors and do not necessarily represent those of their affiliated organizations, or those of the publisher, the editors and the reviewers. Any product that may be evaluated in this article, or claim that may be made by its manufacturer, is not guaranteed or endorsed by the publisher.

## Abbreviations

HCC, hepatocellular carcinoma; HA, hemangioma; ICC, intrahepatic cholangiocarcinoma; CNN, convolutional neural network; ResNet, residual network; MExPaLe model, model fused with minimum extra information about patient and lesion; MEI, minimum extra information; T-test, Student’s *t*-test.
